# Towards bridging the grey digital divide: changes in internet access and its predictors from 2002 to 2014 in Germany

**DOI:** 10.1007/s10433-020-00552-z

**Published:** 2020-03-10

**Authors:** Oliver Huxhold, Elena Hees, Noah J. Webster

**Affiliations:** 1 German Centre of Gerontology, Manfred-von-Richthofen-Straße 2, 12101 Berlin, Germany; 2 Institute for Social Research, University of Michigan, Ann Arbor, MI USA

**Keywords:** Digitalization, Internet use, Ageing, Social exclusion, Social inequality, Cohort

## Abstract

The internet is an indispensable aspect of modern society. It facilitates long distance communication, access to information, health care interventions, as well as multiple opportunities for social participation. Despite increasing pervasiveness of this technology, persistent inequalities exist in who has access to the internet. In particular, older adults lag behind in having internet access, thus putting them at risk for social exclusion. In order to gain a better understanding about the determinants of this *grey digital divide*, the current study contrasts influencing factors of internet access, comparing samples from 2002 to 2014 across age groups (40 to 54 years, 55 to 69 years and 70 to 85 years) using data from the German Ageing Survey (DEAS). Logistic regression confirmed that the likelihood of having internet access was lower with higher age at both time points. However, the percentages of people with internet access grew primarily in the middle and older age groups between 2002 and 2014. Furthermore, being male and having a higher education were both associated with greater odds of internet access. However, gender and education differences in internet access were significantly less pronounced in 2014 in contrast to 2002. Finally, both greater income and cognitive ability were associated with greater odds of internet access, while providing care for a grandchild was significantly associated with internet access only among the oldest age group. In an attempt towards bridging the grey digital divide, the current study serves as a basis for identifying groups mostly affected by this increasingly important form of social inequality.

## Introduction

In an age of rapidly emerging and advancing technologies, the internet has become an essential part of human life. It provides a platform for social interaction as well as facilitates access to information, healthcare services and entertainment. Moreover, considering the prevalence of internet access, a growing number of private and public services are now offered online (Caruso 2018). Thus, being online is becoming increasingly vital in order to stay connected as an engaged participant in our modern society and those without access are in danger of becoming exceedingly excluded from society (United Nations 2014). To better understand differences in internet access in the middle-aged and older adult population, we employ the perspective of a diffusion model of technological innovation (Peres et al. 2010). In particular, we will use this perspective to investigate whether or not factors influencing access to the internet differ across age groups and across historical time.

### The digital divide

According to the diffusion model, the spread of an innovation can be understood as a diffusion process, in which a new technology is introduced to a comparatively small homogenous group—which in most cases belongs to the more socio-economically affluent strata of a population—from which it then slowly spreads out (Peres et al. 2010). In other words, the likelihood of incorporating internet access into an individual’s life style may depend on the prevalence of the use of this technology across the different living contexts that a person is embedded in, such as work, family and one’s personal social network (Peres et al. 2010). Although differences in the accessibility of a newly introduced technology have been demonstrated to disappear over time resulting in a widespread use in the general population (Compaine 2001), internet access to date is still not available to everyone.

“The perceived gap between those who have access to the latest information technologies and those who do not” (Compaine 2001) is called *digital divide*. A number of studies have linked this divide to several negative outcomes including poor health (e.g. Dobransky and Hargittai 2012; Hong and Cho 2016; Hong et al. 2017; Levy et al. 2015) and well-being (e.g. Ihm and Hsieh 2015; Shapira et al. 2007), as well as reduced political knowledge (e.g. Bonfadelli 2002; Wei and Hindman 2011). Furthermore, considering the growing omnipresence of the internet, inequalities in access to and use of internet technologies have become one of the most prominent forms of social inequality with a major influence on life opportunities (Robinson et al. 2015; Warren 2007). Internet adoption, access and overall use can be referred to as a *first-level digital divide,*^1^ while internet skills and literacy mark further differences, already tapping into a *second level digital divide* (Friemel 2016).

As the first-level digital divide is persistent among some groups (Friemel 2016), it is important to gain a thorough understanding of the factors influencing the likelihood of having internet access in order to determine the best approaches and strategies to counteract this divide. In line with the diffusion model, education and income have been found to play a significant role when it comes to internet adoption (Anderson and Perrin 2017; Demoussis and Giannakopoulos 2006; van Deursen and van Dijk 2015). Moreover, general use of the internet may be linked to gender, as females tended to report less use compared to men (van Deursen and van Dijk 2015). This gender gap has been explained as a product of persisting socio-economic inequalities between men and women (van Deursen and van Dijk 2015). However, from a diffusion perspective, gender differences in internet access may also be the consequence of differential exposure to the technology.

### The grey digital divide

One group still experiencing marginalization when it comes to the first-level digital divide are older adults (Friemel 2016). This inequality has been referred to as the *grey digital divide* (Morris 2007; Morris et al. 2007). Similar to gender differences, the grey digital divide may not be a mere consequence of age but could be driven by age group differences in gender ratios, education and income. This effect could be seen as a *double jeopardy* as people’s chances to have internet access might not only be affected by increased age, but also by other socio-demographic variables at the same time. Thus, taking into account demographic changes resulting in an accelerated growth of the older adult population, identifying underlying factors that specifically limit engagement with the internet in later life is equally important. This information can help develop policies and targeted interventions to counteract expansive social inequalities in internet access and use. In this sense, one factor that may limit internet use in older adults in particular could be age-related declines in cognitive ability. Senescent changes in fluid intellectual abilities such as processing speed have been shown to be able to seriously limit the ability to learn (Singer et al. 2003). Thus, for older adults, who did not grow up with the internet, cognitive decline might determine whether or not they are able to access the internet and make use of it. Further, assuming the digital divide is a matter of contextually driven exposure to technology (i.e. the diffusion perspective), it is necessary to also examine possibilities to foster an increase in exposure. Besides direct socio-demographic influences on internet access, grandchildren have been identified to play an important role in facilitating computer use among older adults (Evans 2010). Spending time with grandchildren likely expands grandparents’ exposure to technology and might point towards intergenerational learning as a possible solution in bridging the digital divide. This suggestion also matches the significance of encouragement on internet use by individual social networks as discovered by Friemel (2016).

### Differences in factors influencing internet access over time

Contrasting influencing factors of internet access across age groups may only be a first step for uncovering potential routes to bridge the grey digital divide. As the diffusion model assumes, technologies slowly spread from homogenous groups of early users to adjacent subgroups in the population (Peres et al. 2010). Thus, examining whether or not factors influencing internet access have changed in importance over time may facilitate a greater understanding of mechanisms underlying the spread of internet access. For example, existing social disparities are reflected and possibly reinforced within the digital divide (Robinson et al. 2015). In this sense, previous research has found a generational explanation of the digital divide, in which different birth cohorts were exposed to the internet at different points in their life course, which may have an even more important influence on the adoption of this technology than the exclusive effects of age itself (Gilleard and Higgs 2008). Furthermore, as argued above, old age can be considered a time of elevated risk, in which pre-existing socio-demographic differences exacerbate the grey divide in access to the internet. Thus, it is important to examine historical differences in socio-demographic factors of internet access in different age groups. A specific emphasis in this regard will be placed on the influence of financial factors. Overall, the longer technologies are in use, and they tend to decrease in cost as prices usually show a diffusion-based pattern of firstly increasing and then decreasing over a longer time span (Mahajan et al. 1993). Thus, it seems important to investigate whether or not the limiting factor of financial assets has been decreasing in recent years. There have been a number of initiatives working towards increasing the user friendliness of various technologies used to access the internet (de Jong and Lentz 2006; Jiang et al. 2000; Nykanen 2008; Wimmer and Holler 2002). Therefore, cognitive ability may become less important for internet access at later time points.

### The current study

Given the rapid rate of change in the adoption of technology (Anderson and Perrin 2017), in order to understand the nature of the digital divide, it is important to not only examine its determinants at a fixed date but also across time points. Although previous research has already identified several determinants of internet accessibility, it remains equivocal how observed inequalities become less pronounced or are enhanced through the course of digitalization (Robinson et al. 2015).

The purpose of the current study is to contrast factors associated with internet access across time points and age groups. In particular, we will contrast differences in access to the internet across 12 years from 2002 to 2014 and examine how these historical changes differ across age groups, between genders and groups of people with varying educational attainments. In a second step, we examine whether or not the limiting influences of financial assets (e.g. income) and cognition as well as the potentially facilitating influence of caring for grandchildren have changed across historical time (i.e. from 2002 to 2014). To address the latter set of questions, we will employ multi-group structural equation analyses that will allow us to directly compare the predictor strength of factors across the two time points in three age groups (40 to 54 years, 55 to 69 years and 70 to 85 years). This age sub-division is essential as previous research shows the digital divide to be disappearing between young and middle-aged adults while those age 65 and older continue to access/use the internet significantly less with a rather exponentially growing negative relation between internet use and age for seniors who are age 70 and older (Friemel 2016).

Overall, we expected a more widespread access to the internet in 2014 than in 2002. Since this wider spread is associated with diffusion to different strata in the population, we assumed that demographic characteristics would play a lesser role in 2014 than in 2002. Finally, given reducing costs of internet access and increasing user friendliness of associated technologies in 2014 in comparison with 2002, we hypothesized that the limiting influences of income and cognitive ability in older adults would be smaller in 2014 than 2002.

Our analyses were led by the following specific hypotheses:

#### **H1A**

Access to the internet will be lower at a higher age at both time points.

#### **H1B**

As a consequence of wider spread internet use, age differences will be smaller in 2014 than in 2002.

#### **H2A**

Being male and having higher educational attainment will be associated with a higher likelihood of internet use.

#### **H2B**

Both gender and educational differences will be smaller in 2014 than in 2002.

#### **H3A**

More income, higher cognitive ability and spending time with grandchildren will be associated with a higher likelihood of internet use.

#### **H3B**

The limiting effect of income and cognitive ability will be smaller in 2014 than in 2002.

Historical changes in the predictor strength of caring for grandchildren were analysed in an exploratory manner.

## Method

### Data

We analysed data from the German Ageing Survey (DEAS) provided by the Research Data Centre of the German Centre of Gerontology (DZA) (Engstler and Schmiade 2013). DEAS is a representative survey of adults between the ages of 40 and 85 living in private households in Germany. The DEAS is funded by the Federal Ministry for Family Affairs, Senior Citizens, Women and Youth (BMFSFJ).

#### Participants

The baseline sample of 2002 consisted of 3084 participants of which 2756 answered the item assessing internet access. The 2014 sample consisted of 6002 participants of which 4248 participants responded to the item assessing internet access. Consequently, across the two samples the effective sample size utilized in this study was *N* = 7004.

### Measures

#### Dependent variable

Internet access was measured with an item included in the DEAS written *Drop*-*Off* questionnaire asking participants if they had access to the internet and providing three possible answers: *No*, *Yes, at home* or *Yes, at work*. The last two answers were summarized resulting in a dichotomous variable measuring access to the internet (0 = no access; 1 = have access).

### Determinants of internet access

#### Age

Age was converted to a categorical variable for the analysis consisting of a younger age group (40 to 54 years), a middle age group (55 to 69 years) and an old age group (70 to 85 years). For the analysis, we created two dummy variables, middle age (0 = No; 1 = Yes) and old age (0 = No; 1 = Yes). Our youngest age group served as the reference group.

#### Time

Time was entered as a dummy variable in the analyses (0 = 2002; 1 = 2014).

#### Education

Educational attainment was based on the internationally comparable ISCED classification (United Nations 2006). Participants were divided into three education groups: low, medium and high educational attainment. For the analyses, we created two dummy variables, medium (0 = No; 1 = Yes) and high (0 = No; 1 = Yes). People with low education served as the reference group.

#### Gender

Gender was designated as male or female according to the information of the registration office or participants’ indication in the interview and included as a dummy variable in the analysis (0 = female; 1 = male).

#### Income

Since a person’s financial situation strongly depends not only on his or her own income but also on their household arrangements (e.g. partner’s income, number of people living in the household), we used the OECD (weighted) net equivalised household income as an indicator of available income (OECD 2019; Strauß and Ebert 2013). In a final step, the variable was z-standardized.

#### Cognitive ability

Outcomes on the digit symbol substitution subtest of the Wechsler Adult Intelligence Scale (WAIS; Wechsler 1981) were included in our models. The Digit Symbol Substitution test is used as a measure of cognitive-perceptual speed (Lindenberger et al. 1993). Previous research has shown that cognitive speed mediates negative age differences in knowledge, reasoning, memory and fluency (Lindenberger et al. 1993). Thus, the Digit Symbol substitution subtest can be used as a valid indicator of overall cognitive abilities among older adults. Cognitive ability was introduced into the statistical models as a z-standardized variable.

#### Care of grandchildren

Participants with grandchildren were asked if they take care of their grandchildren. The item was included as a dummy variable in the analysis. Participants, who indicated taking care of one or more grandchildren, got coded with a 1 on this item; 0 indicating the participant does not have grandchildren or does not take care of them.

### Analyses

For our analyses, we employed a logistic regression design using MPlus (Version 8; Muthén and Muthén 2018). Using a structure equation modelling program for the analyses, allowed us to test our hypotheses with preplanned maximum likelihood model contrasts bearing high statistical power. Furthermore, the full information maximum likelihood (FIML) estimation procedure implemented in MPlus allowed us to reduce parameter bias due to item non-response. Item non-response in our data ranged from zero per cent in gender and age to a maximum of nine per cent with respect to income. Monte Carlo simulation studies have shown that FIML significantly reduces parameter bias due to data missingness as long as the models contain predictors that are associated with the patterns of missing data (Graham 2009). This is the case for our analyses. To test hypotheses 1 and 2, we conducted basic logistic regression analyses in which internet use was regressed on time (one dummy variable) and either age group (two dummy variables) or gender (one dummy variable) or education (two dummy variables) and their respective interaction with time (e.g. middle-age-by-time). To address hypothesis 3, we conducted a multi-group multivariable logistic regression analyses in which we contrasted the effect size of income, cognitive ability and care of grandchildren across six different age-by-time groups (i.e. 40–54 years in 2002, 55–69 years in 2002, 70–85 years in 2002, 40–54 years in 2014, 55–69 years in 2014, 70–85 years in 2014). We reported *χ*^*2*^-differences and *p* values up to the third decimal. Our alpha level was set to .05.

## Results

### Age and time analyses

All effects that were significant in the first step (i.e. age and time) analyses reported in next paragraphs remained significant when examined in a meta-model controlling for all other predictors (i.e. time, age group, education, gender, income, cognitive ability and care of grandchildren). We estimated these effects without other covariates in order to obtain and report unbiased estimates of the percentage of respondents with internet access in the respective groups.

### Time

Overall, the number of participants with access to the internet increased significantly from 29.03% in 2002 to 73.59% in 2014 (Δ*χ*^2^= 1381.29, Δ*df* = 1, *p* = .000).

### Age by time

**Fig. 1:**
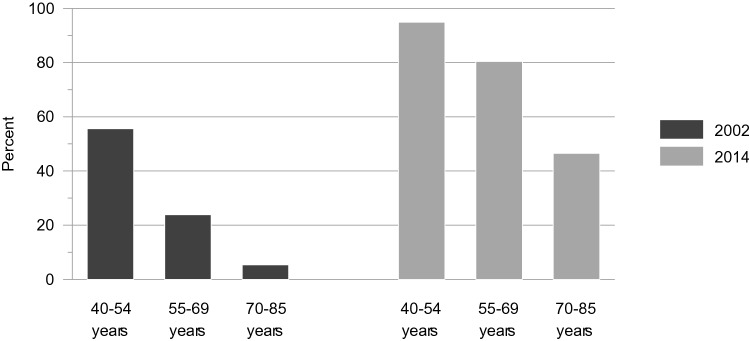
Access to the internet in per cent: age groups by time

In support of hypothesis 1A, the middle and the older age groups reported significantly less access to the internet at both time points (55–69 years: Δ*χ*^2^= 201.83, Δ*df* = 1, *p* = .000; 70–85 years: Δ*χ*^2^= 602.60, Δ*df* = 1, *p* = .000). The middle-age-by-cohort interaction (Δ*χ*^2^= .62, Δ*df* = 1, *p* = .433) and the old-age-by-cohort interaction (Δ*χ*^2^= .01, Δ*df* = 1, *p* = .938) were both not significant. However, the proportional gain in internet access was slightly larger in these groups compared to the 40–54-year-old group (Δ*χ*^2^= 368.93, Δ*df* = 1, *p* = .000). This indicates the percentages of people with internet access grew by a larger factor across 12 years in the middle and the older age group than in the youngest age group. These findings indicate support for hypothesis 1B. The results are displayed in Fig. 1.

### Gender by time

**Fig. 2:**
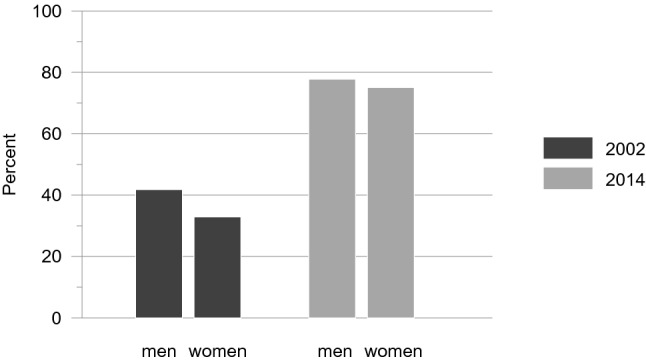
Access to the internet in per cent: gender by time

Overall, men had greater odds of having access to the internet compared to women (Δ*χ*^2^= 20.56, Δ*df* = 1, *p* = .000). The gender difference was significantly larger in 2002 than in 2014 (Δ*χ*^2^= 4.52, Δ*df* = 1, *p* = .034). Averaged across all age groups in 2002, the gender difference amounted to 8.90%. In 2014, only 2.69% more men than women reported having access to the internet. A posteriori test revealed that gender differences were not significant in 2014 (Δ*χ*^2^= 3.07, Δ*df* = 3, *p* = .381). These results provide support for hypotheses 2A and 2B that gender differences have markedly decreased across time. The results are displayed in Fig. 2.

### Education by time

**Fig. 3:**
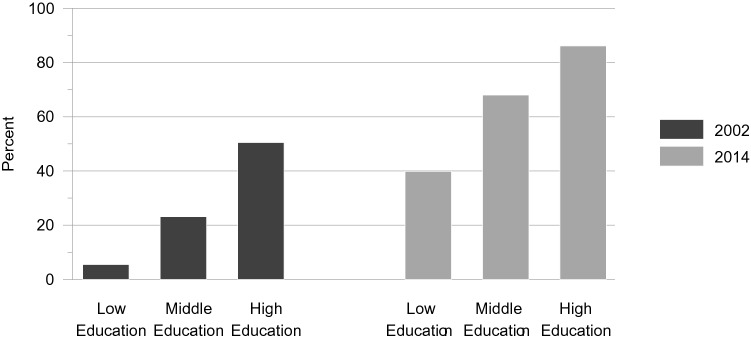
Access to the internet in per cent: education by time

The medium and higher education groups had greater odds of having internet access than the lower education group (medium education: Δ*χ*^2^= 75.00, Δ*df* = 1, *p* = .000; higher education: Δ*χ*^2^= 275.64, Δ*df* = 1, *p* = .000). Thus, this part of hypothesis 2A was confirmed. The medium-education-by-cohort interaction was negative and marginally significant (Δ*χ*^2^ = 3.48, Δ*df* = 1, *p* = .062), and the higher-education-by-cohort interaction was negative and significant (Δ*χ*^2^= 5.66, Δ*df* = 1, *p* = .017). The proportional gain in internet access was slightly lower in the middle and higher education groups compared to the lower education group (Δ*χ*^2^= 72.88, Δ*df* = 1, *p* = .000). Thus, the percentages of people with access to the internet grew by a larger factor across 12 years in the lower education group than in the groups with medium and higher education. Thus, this aspect of hypothesis 2B was confirmed. The results are displayed in Fig. 3.

### Multi-group analyses

Predictors of internet access were successively introduced in the multi-group model. Income was included at first, followed by cognitive ability. Finally, care of grandchildren was introduced into the model. Statistical modelling of each variable followed a series of planned model contrasts. First, it was evaluated whether or not there was a main effect of the predictor. Second, it was examined whether or not the predictor strength varied across time points and age groups. The third and final step consisted of testing a model in which all parameters were freely estimated to explore whether or not there were any significant age -group-by-time combinations. Free estimation, however, did not significantly increase the model fit. Therefore, the results of this contrast are not reported.

#### Income predicting internet access

Over and above the effects of education and gender, income was positively and significantly related to internet access (Δ*χ*^2^= 446.92, Δ*df* = 1, *p* = .000). The predictive strength of income did not differ significantly across time (Δ*χ*^2^= .25, Δ*df* = 1, *p* = .615) nor across age groups (Δ*χ*^2^= 3.57, Δ*df* = 2, *p* = .168). Higher income—as indicated by a salary of one SD or more above the average—was associated with greater odds of having internet access that was 2.94 times greater than for people whose household income was around the average of their respective age-by-cohort group. This association was the same strength in 2002 as in 2014 and across all three age groups.

#### Cognitive ability predicting internet access

Over and above the effects of education, gender and income, cognitive ability was significantly associated with internet access (Δ*χ*^2^= 49.13, Δ*df* = 1, *p* = .000). The predictive strength of cognitive ability significantly differed across time (Δ*χ*^2^= 12.38, Δ*df* = 1, *p* = .000) but not across age groups (Δ*χ*^2^= 4.05, Δ*df* = 2, *p* = .132). Higher cognitive ability was positively associated with greater odds of internet access. However, the strength of this association decreased across time. In 2002, an individual scoring one standard deviation above the average of their age-by-cohort group had a 1.91-times greater odds of having access to the internet than people of average cognitive ability. In 2014, this odds ratio was still significant but reduced to 1.27.

#### Caring for grandchildren predicting internet access

Over and above the effects of education, gender, income and cognitive ability, caring for a grandchild or grandchildren was overall not significantly related to internet access (Δ*χ*^2^= .00, Δ*df* = 1, *p* = .950). The predictive strength of caring for grandchildren also did not differ significantly across time (Δ*χ*^2^= .70, Δ*df* = 1, *p* = .404). The strength of the association did, however, significantly differ across age groups (Δ*χ*^2^= 9.27, Δ*df* = 2, *p* = .010). A post hoc test revealed that the association between caring for grandchildren and internet access could be set to zero for the younger (40 to 54 years) and middle-age group (55–69 years) without a significant loss in model fit (Δ*χ*^2^= 1.24, Δ*df* = 3, *p* = .743). Among those, who were greater than 70 years of age, however, caring for grandchildren increased the odds of having internet access by 1.47 times irrespective of the time of measurement.

In sum, hypothesis 3A was confirmed, while hypothesis 3B was only true for cognitive ability with the influence of income staying the same across time points. Income, cognitive ability and spending time with grandchildren were significantly related to the odds of having access to the internet. However, the association between cognitive ability and internet access decreased significantly over time and the effect of caring for grandchildren was only significant among the oldest age group.

## Discussion

Our results are consistent with a diffusion perspective of the spread of a new technologies (Peres et al. 2010) across the German population 40 years and older. In 2002, access to the internet was more widely spread in a population that was relatively young, male and highly educated. Coinciding with an overall growth in the prevalence of internet access in the following 12 years, women, people with lower education and older adults showed a disproportionally strong increase in comparison with the rest of the population age 40 and older. Thus, socio-demographic differences in access to the internet and consequently social inequalities with respect to the digital divide have been somewhat reduced over this 12-year time period.

Nevertheless, although gender differences disappeared in the observed time frame, the “grey divide” was still present in 2014. Even then, adults over the age of 70 had only half the odds of having access to the internet when compared to those aged 40 to 54. Moreover, educational differences also persisted across the two time points. Thus, the grey divide may in essence create for some people a double jeopardy situation in the sense that the combination of being older and of a lower educated group additively increases inequality in having access to the internet. For example, bearing in mind the increasing prevalence of virtual health care services (Ekeland et al. 2010) and considering at the same time the comparatively large prevalence of multi-morbidity and functional limitations in lower educated older adults (Schöllgen et al. 2010) does strongly speak for the necessity of developing interventions with the purpose of providing internet access that are tailored to this specific group. An example of this is a program being evaluated in the United States Veterans Administration whereby Veterans with geographic, clinical or social barriers to receiving onsite care were given tablets to facilitate and enhance the provision of health care services (U.S. National Library of Medicine 2017). Not-having access to the internet is already linked to lower levels of health and well-being (e.g. Hong et al. 2017; Ihm and Hsieh 2015). Therefore, without implementing public interventions, the increasing digitalization may further reinforce and exacerbate health disparities linked to social inequalities (Robinson et al. 2015).

Some ideas how these interventions can address disparities in access to the internet can be gained from this study’s results. Low income was identified as a limiting factor of obtaining access to the internet at both time points with no indication of decreasing importance over time. Given the rising importance of having access to the internet for active engagement in society (Friemel 2016), state subsidy might be a viable route to decrease digital social inequalities. Furthermore, to our knowledge this is the first study showing the association of fluid cognitive abilities and internet use over and above the effects of education. In younger age groups, low cognitive ability may not pose a problem for acquiring access to the internet for large parts of the population, although from a perspective of social inclusion, developing interventions specifically targeting younger people with learning difficulties may also be advisable. However, fluid cognitive abilities demonstrate marked declines with ageing potentially leading to strong learning difficulties among the oldest-old (Singer et al. 2003). In this study, cognitive ability showed a decreasing influence on internet accessibility over time, which might indicate that internet applications became more user friendly over the 12 year period observed. Despite this, the effect of cognition is still present and substantial even in 2014. Thus, providing hardware and online services which are easily accessible without the need for a specific skill set might be a crucial strategy to decrease barriers to gaining access to the internet for older adults. This may be especially true given the comparatively high possibility of mild cognitive impairments at older ages (Hänninen and Soininen 1997).

Finally, our analyses provided some indirect indication that social contagion (i.e. the spread of specific behaviours in a population through social contact; Christakis & Fowler 2013) might be an important mechanism for the dispersion of internet use in older populations. Older adults, who spent time with their grandchildren, were more likely to have access to the internet than their same age counterparts, irrespective of education, income and cognitive ability. Thus, future interventions could potentially employ the mechanism of social contagion and more specifically intergenerational ties. Previous research has shown health-related behaviour (e.g. the use of complementary medicine) to be influenced by social networks (Goldman and Cornwell 2015). The key factor within this connection is the extent to which one sustains social ties to people who are not connected with each other, which is referred to as network bridging. Identifying and educating people about the internet, who have bridging positions in social networks of older adults, could potentially increase intervention effectivity. This is due to people in bridging positions having a comparatively higher likelihood for acting as multipliers or influential agents of change in a network. Basically, such an intervention may further the potential for social interaction within existing real-life networks by digital means.

## Limitations and outlook on future studies

Although the current study provides a focused overview on factors determining internet access among older adults, there are a few limitations to be considered which might point towards gaps in the literature worth investigating in future research.

The most current data we used for this study were collected in 2014. Although the field of ICT is characterized by rapid changes within recent years, we argue that the results from this study are still applicable. Even though the number of people with access to the internet may have increased, we assume that limiting and facilitating factors for older adults are still salient. However, it is important to stay informed about current developments with respect to social inequalities in internet access, which can take many forms. Thus, future research should continue doing similar analyses contrasting influencing factors of internet access across current time points and social groups.

In a related vein, the data set at hand did not include information about individual psychological factors that may work as mediating influences of the more distal factors examined in this study. Lack of confidence regarding internet use has been shown to be a prominent factor hindering engagement in internet activities, especially among older adults (Hunsaker and Hargittai 2018). Thus, examining interactions between having a lack of confidence and the limiting factors observed in this study (e.g. demographic differences, socio-economic status and cognition) could provide further insight into concrete individual-level mechanisms that lead digital inequalities at the societal level. Similarly, past experiences with internet technology at the work place before retirement could be taken into account to differentiate more clearly between processes related to ageing and cohort differences due to past exposure and their relationship to internet access. Unfortunately, such information was not available in the data set at hand.

Generally, as previously mentioned, the digital divide can be defined on various levels; starting with internet access and general use marking a first digital divide as investigated in the current study, but going further into subsequent divides such as specific types or reasons for internet use dependent on skills and digital literacy. The current study solely refers to the first-level digital divide by examining internet access. Although our study found that this divide still exists, it also shows that societal differences in internet access have become smaller over time. As the first digital divide continues to shrink, future research examining specific types of internet use as well as related skills becomes indispensable. Looking at current developments such as e-government (Gasova and Stofkova 2017), the increasing popularity of extracting health information from the internet (Hong and Cho 2016) or making use of online consulting (Marshall et al. 2018), the internet becomes a necessity in order to stay an engaged, informed and a self-determined citizen. Accordingly, inclusiveness by bridging the digital divide is one of the main goals adopted by the Connect 2020 Agenda of the International Telecommunication Union (ITU), the United Nations specialized agency for ICTs (United Nations 2014). Nevertheless, some other forms of internet use such as entertainment may be of less interest in terms of social participation but still very important for overall well-being. Thus, identifying who uses the internet and for what specific purposes is important in order to target the overcoming of social disparities.

In summary, even though there is room for further research on determinants and remedies of the grey digital divide, the current study determined groups mostly affected by this divide. These findings might serve as a starting point towards further identifying possible resources for interventions tailored for specific vulnerable subgroups.

## Footnotes

1 Within the DEAS 2014 sample, only 1.2% of all people who have access to the internet do not use it for any of the listed purposes in the Drop-off questionnaire (contacting friends and family, searching for new social contacts, searching for information, banking, entertainment, shopping, creating own content). As this is an insignificant fraction and there might be internet uses we did not consider, we believe it is justified to talk about access and use interchangeably.
